# Circulating Dynamics of SARS-CoV-2 Variants between April 2021 and February 2022 in Turkey

**DOI:** 10.1155/2022/4677720

**Published:** 2022-10-15

**Authors:** Murat Sayan, Ayse Arikan, Murat Isbilen

**Affiliations:** ^1^Kocaeli University, Research & Education Hospital, PCR Unit, Kocaeli 41380, Izmit, Turkey; ^2^Near East University, DESAM Research Institute, Nicosia 99138, Northern Cyprus, Mersin 10, Turkey; ^3^Near East University, Department of Medical Microbiology & Clinical Microbiology, Nicosia 99138, Northern Cyprus, Mersin 10, Turkey; ^4^Kyrenia University, Department of Medical Microbiology & Clinical Microbiology, Kyrenia 99320, Northern Cyprus, Mersin 10, Turkey; ^5^Acibadem Mehmet Ali Aydinlar University, Graduate School of Health Sciences, Department of Biostatistics & Bioinformatics, Istanbul 34752, Turkey

## Abstract

The diagnosis of new variants and monitoring their potential effects on diagnosis, therapeutics, and vaccines by genomic sequencing is essential to manage global public crises. In the current study, spike-genome next-generation sequencing was generated from 492 SARS-CoV-2 isolates to evaluate the mutations in Turkey from April 2021 to February 2022. The variant analysis was performed using (Coronavirus Antiviral and Resistance Database (CoV-RDB) by Stanford University). We revealed that the lineages Alpha (B.1.1.7), Beta (B.1.351), Delta (B.1.617.2), Eta (B.1.525), variant of interest (VOI), lota (B.1.526), Zeta (P.2), Omicron (B.1.1.529), and Omicron BA.1 (B.1.1.529.1) were in the circulation in Turkey during the given period. The most common lineages were B.1.1.7, B.1.617.2, B.1.1.529, and B.1.1.529.1 SARS-CoV-2 variant circulation in Turkey seems highly heterogenetic; however, quite similar to the global epidemiologic analysis. The existence of globally circulating variants in the same chronological order in Turkey can be a guide for precautions, treatment, and vaccine options to be taken in the future.

## 1. Introduction

The pandemic character of the coronavirus disease 2019 (COVID-19) illness continues, with 550 million people getting infected and 6 million deaths [1]. The severe acute respiratory syndrome coronavirus-2 (SARS-CoV-2), belonging to the *Coronaviridae* family, is a single-stranded, positive-sense RNA virus. Due to the flexibilization of activities in various countries, various variants of concerns (VOCs) and VOIs has been emerged, changing the panorama of the evolution of the virus so that a more intense mutation rate can indeed be observed [2, 3]. Studies have demonstrated the negative impact of these variants on transmission, vaccines, and therapeutics [2–4]. As these variants have also spread globally, they have generated significant public health worldwide [5]. Currently, Omicron (B.1.1.529) lineages continue to be the circulating dominant variants of concern (VOCs) as it has been reported to be more contagious, despite lower disease severity than other lineages [6–8]. While the proportion of BA.2 lineage declines, BA.5 and BA.4 lineages have been reported to have been detected in 62 and 58 countries, respectively [9].

The first COVID-19 cases in Turkey were reported on March 9th, 2020. Since then, more than 15 million and approximately 100.000 deaths have been reported in Turkey [1]. According to the confirmed cases reported, it is noteworthy that there are three different periods in which the number of cases increased and peaked in Turkey. After the first cases were reported during Spring 2020, the number peaked in the first week of December 2020 (219.546 confirmed cases with 1.494 deaths) (the first wave). The second peak was Spring 2021, with 414.312 confirmed cases and 2.493 deaths (the second wave). Between July 2021 and December 2021, the number of confirmed cases/per day ranged around 140.000, with approximately 1.500 deaths/per day. The third phase was observed when the number of cases started to increase again in July 2021; since then, it peaked in January 2022 (712.091 confirmed cases with 1.922 deaths) (the third wave). At the end of February 2022, the number of subjects decreased, and this decrease continues in Spring [10].

Dissemination and evolution of new strains should be monitored to understand better, these genetic changes' effect on the virus' transmission rate and its impact on vaccines and therapeutics. Whole-genome sequencing and recently becoming more popular virtual phenotyping methods are powerful tools for molecular epidemiological analysis [11]. The study aimed to clarify the genomic diversity of SARS-CoV-2 strains circulating in three main cities of Turkey during five different periods of the pandemic.

## 2. Materials and Methods

### 2.1. Sampling

From April 2021 to February 2022, 492 SARS-CoV-2 strains were analyzed in the study. One hundred and forty-three strains in April 2021, 187 strains in August 2021, 28 strains in September 2021, 32 strains in December 2021, and 102 strains isolated in February 2022 were involved in the study. The strains were isolated from metropolitan cities, Istanbul, Ankara, and Kocaeli from COVID-19 cases in Turkey. These strains were selected as they were identified as potential variations by the mutation-specific SARS-CoV-2 polymerase chain reaction (PCR) screen.

### 2.2. SARS-CoV-2 RNA Isolation and Diagnosis

Viral RNA was extracted from the nasal/oropharyngeal swab fluids using the magnetic particle technic on the GeneRotex96 (Tianlong Science, Xi'an, China). The diagnosis of new coronavirus was performed using double gene target RT-qPCR (BioSpeedy, Istanbul, Turkey). N501Y/variant detection PCR kit (BioSpeedy, Istanbul, Turkey), and N501Y, delHV69-70, and E484K multiple mutation detection PCR kit (RTA Laboratories, Istanbul, Turkey) were applied for mutation screening. Positive strains were prepared for sequencing.

### 2.3. Next-Generation Sequencing (NGS)

All the PCR products were purified for the NGS processing with the NucleoFast 96 PCR kit (Macherey-Nagel, Dueren, Germany). The purified product was quantitated by spectrophotometry using Nanodrop N1000 (Thermo Fisher, Wilmington, USA). Nucleic acids with concentrations adjusted to 0.2 ng/ul were sequenced on the Nextera XT (Illumina, CA, USA) sequencing platform.

The spike glycoprotein region located between 21709 and 23193 bps in the SARS-CoV-2 genome was aligned with the SARS-CoV-2 Wuhan Hu-1 isolate (GenBank accession number; MN908947.3). The primer pairs R: 5′-acacctgtgcctgttaaaacca-3′ and F: 5′-gacaaagttttcagatcctcagttttaca-3′. were used for sequencing [12], and sequencing was performed between the 118F–1652R primer region (∼1500 bp) on the Miseq sequencing platform (Illumina, CA, USA). Based on BWA software, all sequencing data were reassigned with Miseq Reporter (https://bio-bwa.sourceforge.net/). The protocol for NGS PCR sequencing was generated as follows: at 45°C for 10 min, at 95°C for 2 min, then for 40 cycles; 95°C for 10 s, 57°C for the 30 s, and 72°C for 30 s.

### 2.4. Data Quality and Variant Calling

The sequenced data were inserted into the reference genome via BWA software [13] and analyzed with base recalibrator and apply for BQSR programs according to the recommendations of the Genome Analysis Toolkit (Broad Institute, Massachusetts, USA). The Genome Analysis Toolkit is used as an open source under a “new or revised” license and a BSD 3 clause and has been reedited to baseline reading quality. The Haplotype Caller program (Broad Institute, Massachusetts, USA) performed variant calling with mapping quality and read depth. Variant quality (QUAL) below 50, 15, and 500 were excluded from the analysis using the Variant Filtration program (Broad Institute, Massachusetts, USA). Sequences of samples for this region were constructed by modifying the mutations detected on the reference genome.

The variant/lineage classification and mutations of the strains were identified using the web tool Stanford University Coronavirus Antiviral and Resistance Database (https://covdb.stanford.edu/sierra/sars2/by-patterns/). All analyzes were performed and recorded one month after the sample collection. However, analyzes were repeated every three months to identify possible altered variants and patterns due to the update to the Stanford database. All circulating variants are analyzed in the Stanford web tool, but the relevant variant may not be detected in circulation despite the analysis. Therefore, variants for which data could not be obtained this way were defined as “not determined.”

### 2.5. Ethical Approval

This study received ethical approval from the Scientific Research Ethics Committee of the Near East University with the decision of 1383 NEU/2021/93. As the study was retrospectively conducted, no informed consent form was required.

## 3. Results

Between April 2021 and February 2022, 492 SARS-CoV-2 strains have been sequenced. Among these strains, 414 (64%) were identified as SARS-CoV-2 variants, while 78 (16%) were reported as wild type (WT). Circulating variants of SARS-CoV-2 in Turkey were evaluated using a web tool that provides similar results to the phylogenetic analysis in the current study [11]. Based on this analysis, between April 2021 and February 2022, 7 different lineages and a sublineage were reported among 414 sequences.

**Figure 1:**
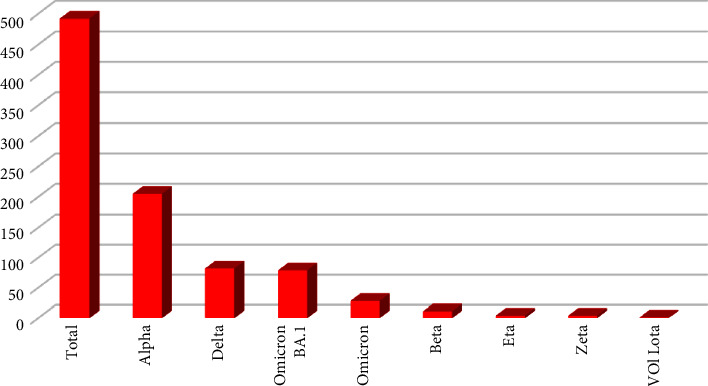
SARS-CoV-2 variant distribution in Turkey.

In April 2021, 5 different lineages including Alpha (B.1.1.7) (*n* = 100, 89%), Beta (B.1.351) (*n* = 5, 4%), Delta (B.1.617.2) (*n* = 4, 4%), Eta (B.1.525) (*n* = 2, 2%), and VOI lota (B.1.526) (*n* = 1, 1%) were identified. The distribution of determined SARS-CoV-2 variants in Turkey is represented in Figure 1. While the dominancy of Alpha variant continued in August 2021 (*n* = 105, 75%); other variants including Beta, Delta, Eta, and Zeta were reported in the rates *n* = 6, 4%, *n* = 23, 16%, *n* = 2, 2%, and *n* = 4, 3%, respectively. Alpha's most frequent S gene mutation was ∆69–70, ∆144, and N501Y. The B.1.351 was characterized by only the D80A, D215G, ∆241–243, K417N, E484K, and N501Y mutation pattern, while B.1.617.2 was mainly characterized by T95I, G142D, Δ156–157, R158G, L452R, T478K, D614G, P681R, and D950N.

**Figure 2:**
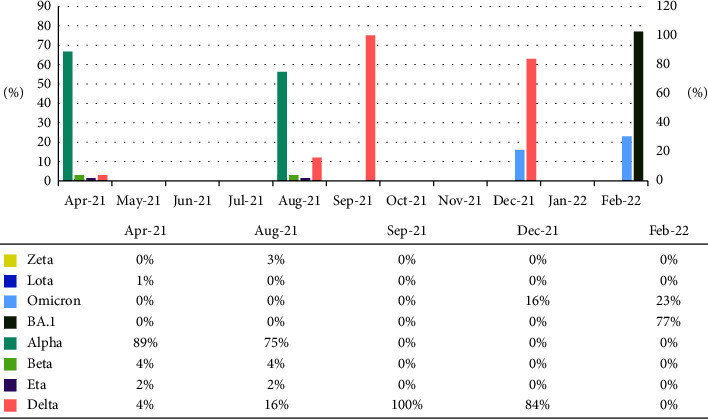
Distribution of SARS-CoV-2 lineages in months in Turkey.

**Figure 3:**
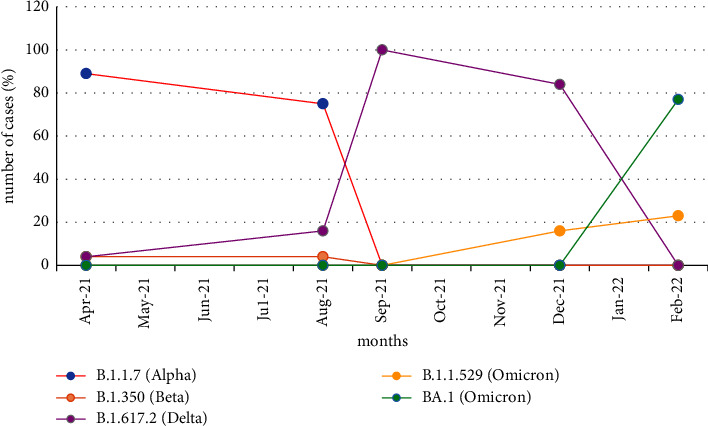
SARS-CoV-2 variant peaks and waves by months.

In September 2021, B.1.617.2 lineage (*n* = 28, 100%) were the dominant in Turkey. Other lineages were not detected among the sequenced strains. In Turkey, Delta was circulating in December 2021 with a higher rate (*n* = 27, 84%) while a new strain named Omicron (*n* = 5, 16%) was reported for the first time in the same month, mainly with the A67V, Δ69–70, T95I, G142D, Δ143–145, N211I, Δ212, 5214R_EPE, G339D, S371F, S373P, S375F, K417N, N440K, G446S, S477N, T478K, E484A, Q493R, G496S, Q498R, N501Y, Y505H, D614G, H655Y, N679K, P681H, N764K, D796Y, N856K, Q954H, N969K, and L981F mutation pattern. B.1.1.529 (Omicron) was spread rapidly and dominated in February 2022. In the period from December 2021 to February 2022, Omicron BA.1 subtype was reported in 77% and was characterized by the mutation pattern: S45X, A67V, Δ69–70, T95I, G142D, Δ143–145, N211I, Δ212, R214R_EPE, G339D, R346K, S371F, S373P, K417N, N440K, G446S, S477N, T478K, E484A, Q493R, G496S, Q498R, and N501Y, while Omicron was detected in 23%. The SARS-CoV-2 lineages circulating in Turkey and spike mutations are represented in Table 1.  Figure 2 represents the distribution of SARS-CoV-2 lineages in months in Turkey. Variants not determined during the study period are indicated as zero in Figure 2. Moreover, Figure 3 demonstrates the peaks and waves of VOIs by month.

The B.1.1.7 was relatively dominant during the five different periods in April and August 2021. Although B.1.617.2 had been in circulation since April 2021, Delta dominated during September and December 2021. Since the first emergence of Omicron in December 2021, Omicron and its subtype BA.1 became dominant in Turkey in February 2022 due to its rapid spread and increased transmissibility.

## 4. Discussion

Undoubtedly, molecular epidemiological analysis is invaluable for monitoring the evolution of new viruses and understanding the impact of emerging viruses on diagnostic kits, therapeutic agents, and vaccines. In the current study, spike-genome sequencing was generated from 492 SARS-CoV-2 isolates to evaluate the mutations in Turkey from April 2021 to February 2022. We revealed that the lineages Alpha (B.1.1.7), Beta (B.1.351), Delta (B.1.617.2), Eta (B.1.525), VOI lota (B.1.526), Zeta (P.2), Omicron (B.1.1.529), and Omicron (BA.1) were in the circulation in Turkey in the time interval. The most common lineages were B.1.1.7, B.1.617.2, B.1.1.529, and B.1.1.529.1. It seems that SARS-CoV-2 variant circulation in Turkey is highly heterogenetic within the given period we examined. For almost 1–1.5 years, four different variants were dominant in Turkey. Following the global spread of SARS-CoV-2 at the beginning of 2020, intercontinental travel was restricted to reduce viral spread. After that, summer travel resumed in many countries in the further phases of the pandemic [14], which might have stimulated the different types of viruses to spread between continents. This also shows us that the PCR diagnosis of SARS-CoV-2 should have high compliance with viral variability.

Alpha (September 2020), Beta (May 2020), Gamma (November 2020), and Delta (October 2020) were previously circulating VOCs, while Omicron (November 2021) is the only VOCs in circulation [5]. According to our retrospective genome analysis, Turkey's variation distribution during the COVID-19 pandemic is similar to the world in chronological order [5]. While lineage B.1.1.7 were documented at the highest rates in April 2021, B.1.351 and P.1 lineages were also reported globally [15]. The data on geographic spread and prevalence of VOCs supports our results as several SARS-CoV-2 lineages were reported in April 2021, while the Alpha dominated during the second wave in Turkey. In a recent study conducted in Turkey between March 2021 and December 2021, B.1.1.7 was dominant in April 2021 [16]. B.1.1.7 was reported as the most common lineage in Pakistan, one of the most populated countries in that month [17]. Similarly, the most common variant during Spring 2021 was reported Alpha in the United States [18]. Alpha is characterized by increased transmissibility, detected as the dominant variant in many countries, and the dominance continued until the summer of 2021 in Turkey and globally [18, 19].

According to the epidemiological reports on COVID-I9, the Beta variant was the second most common variant detected globally after the Alpha variant in August 2021 [20]. Our genome analysis was also similar to global genomic analysis. We reported a lower rate for B.1.350 (Beta) and other variations, including B.1.525 (Eta) and B.1.526 (VOI lota), from early Spring 2021 to late Summer 2021, and observably, their incidence dropped sharply. Since the beginning of autumn, a sharp increase in the rate of the Delta variant has been observed, and it has been determined as the only variant in Turkey. As the pandemic continued, the primary concern of genetic diversity was whether these changes would impact drug and vaccine efficacy. According to the studies, people infected with B.1.617.2 were more likely to be hospitalized or have more severe outcomes than B.1.351 [21]. Delta was characterized by increased transmissibility compared to Alpha (40%–80%) [18]. In September 2021, the global rate of hospital admissions for COVID-19 accelerated sharply due to the Delta wave [22]. The highest rate of hospitalized infection occurred in the United States (*n* = ∼87.000), followed by the United Kingdom (*n* = ∼7.000) and France (*n* = ∼6.000) [21]. Delta became dominant globally until December 2021 [23] and its effect continued in Turkey until then. Our findings showed that Delta was dominant in September–December 2021 in the country. During Delta's dominance, the death rate in Turkey had nearly doubled [10]. The effect of the Delta variant on the rate of deaths demonstrates the importance of continuous monitoring of emerging variants and their potency on antibodies, vaccines, and diagnosis escapes, as variants of SARS-CoV-2 can spread rapidly worldwide.

Specifically, this retrospective epidemiologic analysis revealed that Omicron has been circulating with Delta since December 2021 in Turkey. Since it was first documented in November 2021, B.1.1.529 and its sublineages, including BA.1, BA.2, BA.3, BA.4, and BA.5, were emphasized to be monitored and comparatively evaluated for the virus characteristics [5, 24] because Omicron raises concerns as it may reduce antibodies produced by COVID-19 and the effectiveness of vaccines [25]. Since January 2020, nearly 12 million SARS-CoV-2 genomes have been uploaded to the GISAID data platform [26]. Although the number of submissions of the sequences continues to decrease compared to the previous months, among the lines, Omicron is still the dominant variant circulating globally [27]. According to the data on the geographic spread and the distribution of VOCs, while the rate of the sublineages BA.2 and BA2.12.1 trends to decline, an acceleration was reported in the BA.4 and BA.5 on 13–19 June 2022 [27]. It should be noted that the limitation of surveillance systems, mainly the sequencing capacity of different countries, may also affect these trends.

We only reported the BA.1 sublineage of Omicron in February 2022. However, the rapid spread of sublineages BA.4 and BA.5 makes us think of reaching these variants in Turkey in the future. Although Omicron has been shown to cause milder infections compared to other variants of SARS-CoV-2, it should always be considered that some Omicron sublineages may cause more severe disease than different Omicron varieties [28, 29]. In France, a recent study provided information on the severity of BA.2 compared to BA.1 sublineages of Omicron [30]. Therefore, continuing with the proper surveillance and precaution measures, the rate of the cases could be controlled in Turkey like in other countries.

The existence of globally circulating variants in the same chronological order in Turkey can be a guide for precautions, treatment, and vaccine options to be taken. Heterogenic variant dynamics in SARS-CoV-2 have led to diagnostic and vaccine development processes that we have not experienced before. On the other hand, the diagnosis and escape of vaccine may be possible in SARS-CoV-2 variants. Our findings, therefore, show how necessary viral genomic surveillance is.

## Figures and Tables

**Table 1:** Spike mutation pattern according to lineage and variants of SARS-CoV-2 strains circulating in Turkey.

Lineages	Spike mutation patterns	CoV-RDB, *n* (%)
B.1.1.7/Alpha		205 (42)
	∆69–70, ∆144, N501Y	161 (79)
	∆69–70, ∆144, N501Y, A570D, D614G, P681H, T716I	7 (3)
	∆69–70, S98F, ∆144, and N501Y	6 (4)
	∆144 and N501Y	5 (3)
	∆69–70, ∆144, N501Y, A570D, D614G, P681H, S704L, T716I, and	4 (2)
	N501Y	3 (1)
	∆69–70, ∆144, G181V, and N501Y	3 (1)
	∆69–70, ∆144, V289L, and N501Y	3 (1)
	∆69–70, ∆144, S155R, F374S, and N501Y	3 (1)
	∆69–70, ∆144, S155R, and N501Y	2 (1)
	∆69–70, L141F, ∆144, and N501Y	2 (1)
	∆69–70, ∆142, Y144V, and N501Y	2 (1)
	A67V, ∆69–70, ∆144, and N501Y	1 (0.5)
	∆69–70, ∆144, A260V, and N501Y	1 (0.5)
	∆69–70 and N501Y	1 (0.5)
	S98F, ∆144 and N501Y	1 (0.5)

B.1.351/Beta		11 (2)
	D80A, D215G, ∆241–243, K417N, E484K, and N501Y	6 (100)

B.1.617/Delta		82 (16)
	T95I, G142D, ∆156–157, R158G, L452R, T478K, D614G, P681R, and D950N	27 (33)
	T95I, G142D, ∆156–157, R158G, L452R, and T478K	9 (11)
	G142D, ∆156–157, R158G, L452R, and T478K	7 (9)
	G142D, ∆156–157, R158G, L452R, T478K, D614G, P681R, and D950N	6 (7)
	T95I, G142D, ∆156–157, R158G, L452R, T478K, D614G, P681R, D950N, and E1202Q	3 (4)
	K41X, T95I, G142D, Δ156–157, R158G, L452R, T478K, D614G, P681R, and D950N	3 (4)
	G142D, ∆156–157, R158G, A222V, L452R, and T478K	3 (4)
	T95I, G142D, ∆156–157, R158G, L452R, T478K, D614G, P681R, D950N, A1020V, and	2 (3)
	A222V	2 (3)
	G142D, ∆156–157, R158G, W258R, L452R, T478K, D614G, P681R, and D950N	2 (3)
	T95I, G142D, ∆156–157, R158G, L452R, T478K, D614G, P681R, D950N, and H1159R	2 (3)
	G142D, ∆156–157, R158G, N440T, and L452R	1 (1)
	L452R and N501Y	1 (1)
	T478K	1 (1)
	C136F, ∆144, A222V, ∆242–243, L452R, T478R, and E484Q	1 (1)
	K77T, G142D, ∆156–157, R158G, L452R, and T478K	1 (1)
	T95I, G142D, ∆156–157, R158G, L452R, T478K, D614G, P681R, D950N, and V1228L	1 (1)
	T95I, G142D, ∆156–157, R158G, L452R, T478K, D614G, P681R, D950N, and F1075L	1 (1)
	T95I, G142D, ∆156–157, R158G, L452R, T478K, D614G, P681R, D950N, and V1104L	1 (1)
	T95I, G142D, Δ156–157, R158G, L452R, T478K, D614G, P681R, D950N, and E1202Q	1 (1)
	G142D, ∆156–157, R158G, L452R, T478K, D614G, P681R, D950N, and M1237I	1 (1)
	T95I, G142D, ∆156–157, R158G, A352V, L452R, T478K, D614G, P681R, N777D, and D950N	1 (1)
	G142D, Δ156–157, R158G, T385I, L452R, T478K, D614G, P681R, D950N, and H1159R	1 (1)
	T95I, G142D, ∆156–157, R158G, L452R, T478K, D614G, P681R, D950N, and I850L	1 (1)
	V42X, T95I, G142D, Δ156–157, R158G, E224Q, L452R, T478K, D614G, P681R, A688V, I850L, M869V, and D950N	1 (1)
	V831, T95I, G142D, Δ156–157, R158G, L452R, T478K, D614G, P681R, and D950N	1 (1)
	Δ156–157, R158G, T478K, D614G, P681R, and S1161SAPT	1 (1)

B.1.525/Eta		4 (1)
	A67V, ∆69–70, ∆144, and E484K	1 (25)
	A67V, ∆69–70, ∆144, and N5017	1 (25)
	Q52R, A67V, ∆69–79, ∆144, and E484K	1 (25)
	Q52R, A67V, ∆69–79, ∆144, S22IL, and E484K	1 (25)

P.2/Zeta		4 (0.8)
	D138Y L178I, S477N, A522S, D614G, Q675R, and A845S	2 (50)
	T95I, ∆144, and E484K	1 (50)

B.1.526/VOI lota		1 (0.2)
	F157S and A520S	1 (100)

B.1.1.529/Omicron		28 (6)
	S45X, A67V, Δ69–70, T95I, G142D, Δ143–145, N211I, Δ212, R214R_EPE, G339D, R346K, S371F, S373P, K417N, N440K, G446S, S477N, T478K, E484A, Q493R, G496S, Q498R, N501Y, T547K, D614G, H655Y, N679K, P681H, N764K, D796Y, N856K, Q954H, N969K, and L981F	17 (60)
	A67V, Δ69–70, T95I, G142D, Δ143–145, N211I, Δ212, 5214R_EPE, G339D, S371F, S373P, S375F, K417N, N440K, G446S, S477N, T478K, E484A, Q493R, G496S, Q498R, N501Y, Y505H, D614G, H655Y, N679K, P681H, N764K, D796Y, N856K, Q954H, N969K, and L981F	9 (32)
	V70X, T95I, G142D, Δ143–145, N211I, Δ212, R214R_EPE, G339D, S371F, S373P, S375F, K417N, N440K, G446S, S477N, T478K, E484A, Q493R, G496S, Q498R, N501Y, Y505H, D614G, H655Y, N679K, P681H, N796Y, N856K, Q954H, N969K, and L981F	1 (4)
	MIK, Δ3-50, D531, F55S, L56F, P57T, A67V, Δ69-70, G142D, Δ143–145, N211I, Δ212, R214X_EPE, Q218H, G339D, S371F, S373P, S375F, K417N, N440K, G446S, S477N, T478K, E484A, Q493R, G496S, Q498R, N501Y, Y505H, D614G, H655Y, N679K, P681H, N764K, D796Y, N856K, Q954H, N969K, and L981F	1 (4)

BA.1/Omicron		79 (16)
	S45X, A67V, Δ69–70, T95I, G142D, Δ143–145, N211I, Δ212, R214R_EPE, G339D, R346K, S371F, S373P, K417N, N440K, G446S, S477N, T478K, E484A, Q493R, G496S, Q498R, N501Y, T547K, D614G, H655Y, N679K, P681H, N764K, D796Y, N856K, Q954H, N969K, and L981F	79 (100)

Wild type, no mutation		78 (16)

Total		492 (100)

CoV-RDB: Stanford coronavirus antiviral and resistance database.

## Data Availability

No data were used to support this study.
